# An Atrial Septal Ridge Diagnosed by Transesophageal Echocardiography

**DOI:** 10.14797/mdcvj.1099

**Published:** 2022-04-18

**Authors:** Rowa H. Attar, Amr Telmesani, Nadeen N. Faza

**Affiliations:** 1 Houston Methodist DeBakey Heart & Vascular Center, Houston, Texas, US

**Keywords:** atrial septal ridge, transesophageal echocardiography, transseptal access

## Abstract

A left atrial ridge is an anomaly of irregular fusion between the septum primum and septum secundum.^1^ Aberrant fusion of the septa results in thickened and fibrotic tissue along the region of the fossa ovalis that will occasionally protrude into the left atrium.^2^ The presence of a left atrial ridge has multiple clinical implications due to its close proximity to the fossa ovalis. The location of this uncommon incongruence may make transseptal catheter-based approaches more challenging, underscoring the importance of imaging guidance to determine the ideal transseptal puncture site.

***Figure 1*** shows cardiac images of a 64-year-old female with a history of severe mitral regurgitation, atrial fibrillation, sick sinus syndrome status post pacemaker implantation, pulmonary hypertension, systemic lupus erythematosus, and chronic kidney disease. She was seen by the valve team and underwent a transesophageal echocardiogram (TEE) to determine candidacy for transcatheter edge-to-edge repair of the mitral valve. Two-dimensional biplane imaging of the interatrial septum (IAS) shows a linear structure on the left atrial side of the fossa ovalis. Three-dimensional imaging of the IAS revealed that the structure was consistent with an atrial septal ridge.

**Figure 1:**
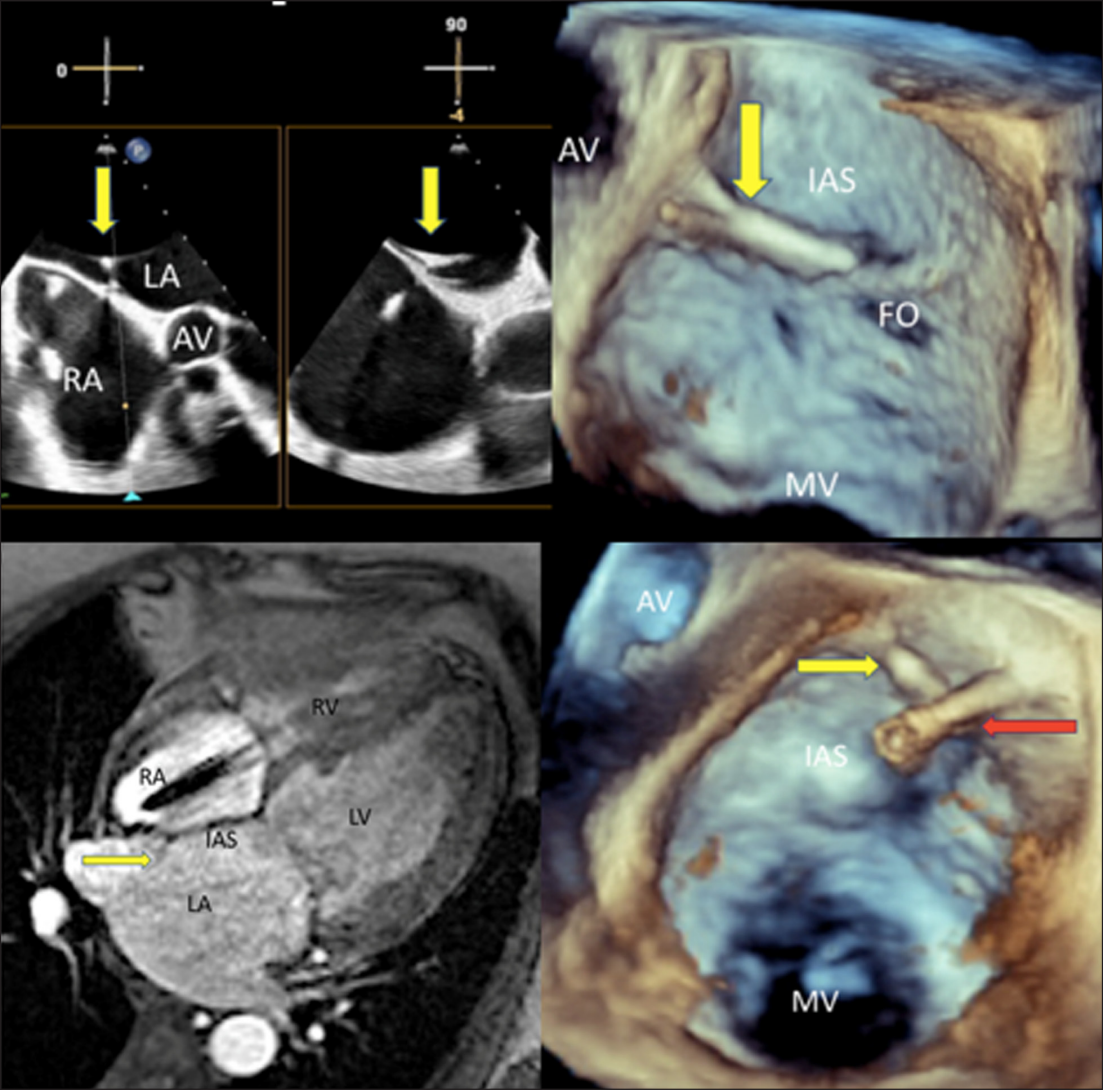
(**A**) Transesophageal echocardiogram (TEE) 2-dimensional (2D) imaging of the interatrial septum with the atrial septal ridge shown by the yellow arrows. (**B**) 3D TEE rotated view of the interatrial septum with the fossa ovalis shown in plane with the ridge (yellow arrow). (**C**) Cardiac magnetic resonance cine SSFP 4-chamber image demonstrating the atrial septal ridge (yellow arrow). (**D**) The MitraClip transcatheter mitral valve delivery system safely traversed across the interatrial septum (red arrow) with the yellow arrow pointing towards the atrial septal ridge. RA: right atrium; LA: left atrium; AV: aortic valve; IAS: interatrial septum; FO: fossa ovalis; MV: mitral valve
